# Supplementary material for: Submerged and Solid-State Fermentation of Spirulina with Lactic Acid Bacteria Strains: Antimicrobial Properties and the Formation of Bioactive Compounds of Protein Origin
Source: Biology (Basel). 2023 Feb 3;12(2):248. doi: 10.3390/biology12020248 (PMC9952912; doi:10.3390/biology12020248)
Supplement: Supplementary file 1 [file biology-12-00248-s001.zip › Supplementary File 1. Analysis of L-glutamic acid (L-Glu) and gamma-aminobutyric (GABA) acids .pdf]

Evaluation of the concentration of L-glutamic acid (L-Glu) and gamma-aminobutyric (GABA) acids in Spirulina samples

An amount of 0.5 g of Spirulina sample was extracted in 50 mL of Milli-Q water for 10 min using an overhead shaker. The samples were incubated for 30 min at 60 °C in a water bath. Then the tubes were cooled down and centrifuged at 4500 rpm for 10 min. A 1 mL aliquot of the supernatant was transferred into 15 mL polypropylene test tubes and diluted with 9 mL of Milli-Q water. Finally, samples were filtered and transferred to a 2 mL autosampler vial. Analysis was performed on a TSQ Quantiva MS/MS coupled to Thermo Scientific Ultimate 3000 HPLC instrument (Thermo Scientific, Waltham, MA, USA). Chromatographic separation was carried out on a Luna Omega Polar C18 (2.1 mm × 100 mm, 3.0 µm) column at 40 °C using an injection volume of 5 µL. The mobile phase consisted of a 0.5 mM ammonium acetate solution in Milli-Q water (eluent A) and methanol (eluent B). A flow-rate of 0.2 mL/min was used. The following gradient conditions were applied: 0.00 min, 1% B (99% A); 1.00 min, 1.0% B (99% A); 6.00 min, 99% B (1% A); 7.50 min, 99% B (1% A); 8.00 min, 1% B (99% A); and 10.00 min, 1% B (99% A). LC-MS interface conditions for the ionization of GABA and L-Glu in the positive ESI mode were as follows: needle voltage + 4500 V; sheath gas 60 Arb; aux gas 25 Arb; sweep gas 5 Arb; ion transfer tube temperature 200 °C; vaporizer temperature 350 °C. The main fragments were identified using the selected reaction monitoring (SRM), with the following ionic transitions: GABA (m/z 104 > m/z 45.151, CE 25.72 V; m/z 104 > 69.165, CE 15.92 V; m/z 104 > m/z 87.36, CE 10.66 V); L-Glu (m/z 148 > m/z 56.05, CE 30 V; m/z 148 > m/z 84, CE 30 V). Method recovery ranged from 59 to 112% for GABA and from 58 to 152% for L-Glu. Method repeatability ranged from 5 to 23% for GABA and from 1 to 20% for L-Glu. The results were obtained in some rounds of experiments on different days.
