# Supplementary material for: Submerged and Solid-State Fermentation of Spirulina with Lactic Acid Bacteria Strains: Antimicrobial Properties and the Formation of Bioactive Compounds of Protein Origin
Source: Biology (Basel). 2023 Feb 3;12(2):248. doi: 10.3390/biology12020248 (PMC9952912; doi:10.3390/biology12020248)
Supplement: Supplementary file 1 [file biology-12-00248-s001.zip › Supplementary File 2. Evaluation of the concentration of biogenic amines (BA) in Spirulina samples.pdf]

Evaluation of the concentration of biogenic amines (BA) in Spirulina samples

Sample preparation and determination of the BAs, including tryptamine (TRP), phenylethylamine (PHE), putrescine (PUT), cadaverine (CAD), histamine (HIS), tyramine (TYR), spermidine (SPRMD) and spermine (SPRM) in Spirulina samples was conducted by following the procedure reported by Ben-Gigirey et al. (1998) [35] with some modifications. Briefly, standard BA solutions were prepared by dissolving known amounts of each BAs (including internal standard) in 20 mL of deionized water. The extraction of BAs in samples (5 g) was done by using 0.4 mol/L perchloric acid. The derivatization of sample extracts and standards was performed using a dansyl chloride solution (10 mg/mL) as a reagent. The chromatographic analyses were carried out using a Varian ProStar HPLC system (Varian Corp., Palo Alto, California, USA) with two ProStar 210 pumps, a ProStar 410 auto-sampler, a ProStar 325 UV/VIS Detector and Galaxy software (Agilent, Santa Clara, California, USA) for data processing. For the separation of biogenic amines, a Discovery ® HS C18 column (150 × 4.6 mm, 5 μm; SupelcoTM Analytical, Bellefonte, Pennsylvania, USA) was used. The eluents were ammonium acetate (eluent A) and acetonitrile (eluent B) and the elution program consisted of a gradient system with a 0.8 mL/min flow-rate. The detection wavelength was set to 254 nm, the oven temperature was 40 °C and samples were injected in 20 μL aliquots. The target compounds were identified based on their retention times in comparison to their corresponding standards.
