# Supplementary material for: Submerged and Solid-State Fermentation of Spirulina with Lactic Acid Bacteria Strains: Antimicrobial Properties and the Formation of Bioactive Compounds of Protein Origin
Source: Biology (Basel). 2023 Feb 3;12(2):248. doi: 10.3390/biology12020248 (PMC9952912; doi:10.3390/biology12020248)
Supplement: Supplementary file 1 [file biology-12-00248-s001.zip › Supplementary File 3. Influence of the analysed factors [lactic acid bacteria (LAB) used for fermentation; duration of fermentation; fermentation conditions] and their interaction on analysed parameters.pdf]

Table S1. Influence of the analysed factors [lactic acid bacteria (LAB) used for fermentation; duration of fermentation; fermentation conditions] and their interaction on lactic acid bacteria viable counts and pH of the Spirulina samples.

Spirulina parameters	LAB	Duration of fermentation	SMF-SSF	LAB × Duration of fermentation	LAB × SMF-SSF	Duration of fermentation × SMF-SSF	LAB × Duration of fermentation × SMF-SSF
			p values				
pH	0.724	0.524	0.066	0.527	0.246	0.124	0.795
LAB viable counts	0.154	0.481	0.067	0.439	0.533	0.284	0.693

LAB – lactic acid bacteria strain used for fermentation; SMF – submerged fermentation; SSF – solid-state fermentation; Factor or factors interaction is significant, when $p \leq 0.05$

Table S2. Influence of the analysed factors [lactic acid bacteria (LAB) used for fermentation; duration of fermentation; fermentation conditions] and their interaction on the concentrations of L-glutamic (L-Glu) and gamma-aminobutyric (GABA) acids in Spirulina samples.

Spirulina parameters	LAB	Duration of fermentation	SMF-SSF	LAB × Duration of fermentation	LAB × SMF-SSF	Duration of fermentation × SMF-SSF	LAB × Duration of fermentation × SMF-SSF
			p values				
GABA	**0.001**	0.868	**0.019**	0.561	**0.011**	0.497	0.503
L-Glu	0.368	0.910	**0.0001**	0.889	0.433	0.937	0.771

GABA – gamma-aminobutyric acid; L-Glu – L-glutamic acid; LAB – lactic acid bacteria strain used for fermentation; SMF – submerged fermentation; SSF – solid-state fermentation; Factor or factors interaction is significant, when $p \leq 0.05$.

Table S3. Influence of the analysed factors [lactic acid bacteria (LAB) used for fermentation; duration of fermentation; fermentation conditions] and their interaction on biogenic amine (BA) content in Spirulina.

Spirulina parameters	LAB	Duration of fermentation	SMF-SSF	LAB × Duration of fermentation	LAB × SMF-SSF	Duration of fermentation × SMF-SSF	LAB × Duration of fermentation × SMF-SSF
			p values				
TRP	**0.001**	**0.021**	**0.0001**	**0.001**	**0.0001**	**0.0001**	**0.0001**
PUT	**0.0001**	**0.0001**	**0.0001**	**0.0001**	**0.0001**	**0.0001**	**0.0001**
CAD	**0.0001**	**0.0001**	**0.0001**	**0.0001**	**0.0001**	**0.0001**	**0.0001**
HIS	**0.0001**	**0.0001**	**0.0001**	**0.0001**	**0.0001**	**0.0001**	**0.0001**
TYR	**0.0001**	**0.0001**	**0.0001**	**0.0001**	**0.0001**	**0.0001**	**0.0001**
SPRMD	**0.0001**	0.370	**0.0001**	0.082	**0.0001**	0.229	**0.020**
SPRM	**0.0001**	0.263	**0.0001**	**0.0001**	**0.0001**	0.001	**0.001**

LAB – lactic acid bacteria strain used for fermentation; SMF – submerged fermentation; SSF – solid-state fermentation; TRP – tryptamine; PHE – phenylethylamine; PUT – putrescine; CAD – cadaverine; HIS – histamine; TYR – tyramine; SPRMD – spermidine; SPRM – spermine; Factor or factors interaction is significant, when $p \leq 0.05$.

Table S4. Influence of the analysed factors [lactic acid bacteria used for fermentation; duration of fermentation; fermentation conditions] and their interaction on diameter of inhibition zones (DIZ) by Spirulina against *Staphylococcus aureus*.

Spirulina parameters	LAB	Duration of fermentation	SMF-SSF	LAB × Duration of fermentation	LAB × SMF-SSF	Duration of fermentation × SMF-SSF	LAB × Duration of fermentation × SMF-SSF
			p values				
DIZ	**0.0001**	0.209	0.514	**0.0001**	0.822	**0.0001**	**0.0001**

DIZ – diameter of inhibition zones; LAB – lactic acid bacteria strain used for fermentation; SMF – submerged fermentation; SSF – solid-state fermentation; Factor or factors interaction is significant, when $p \leq 0.05$.
