# Supplementary material for: Submerged and Solid-State Fermentation of Spirulina with Lactic Acid Bacteria Strains: Antimicrobial Properties and the Formation of Bioactive Compounds of Protein Origin
Source: Biology (Basel). 2023 Feb 3;12(2):248. doi: 10.3390/biology12020248 (PMC9952912; doi:10.3390/biology12020248)
Supplement: Supplementary file 1 [file biology-12-00248-s001.zip › Supplementary File 4. Biogenic amines content .pdf]

Table S1. Biogenic amine (BA) concentrations in control Spirulina samples.

Spirulina samples	Fermentation		Biogenic amines concentration, mg/kg							
	Duration, h	Conditions	TRP	PHE	PUT	CAD	HIS	TYR	SPRMD	SPRM
**Control (I)**	-		2.32±0.99	nd	5.43±1.05	0.873±0.098	nd	6.32±1.13	202.3±11.2	11.6±3.2
**Control (II)**	-		2.66/1.02	nd	6.79±1.11	0.939±0.102	nd	7.78±1.23	279.1±12.8	13.8±4.1

Control (I) - Spirulina powder and water mixture, 1:20 w/w; Control (II) - Spirulina powder and water mixture, 1:2 w/w; SSF – solid state fermentation; SMF – submerged fermentation; TRP – tryptamine; PHE - phenylethylamine; PUT - putrescine; CAD - cadaverine; HIS - histamine; TYR - tyramine; SPRMD - spermidine; SPRM – spermine; nd - not determined.
Data are represented as means (n = 6) ± standard error. $^{a-b}$ mean values denoted with different letters, indicates significantly different values between the lines ($p \leq 0.05$).

Table S2. Biogenic amine (BA) concentrations in Spirulina samples.

Spirulina samples	Fermentation		Biogenic amines concentration, mg/kg							
	Duration, h	Conditions	TRP	PHE	PUT	CAD	HIS	TYR	SPRMD	SPRM
***Lacticaseibacillus paracasei* No. 244**	24h	SMF	4.77±0.8a	nd	5.84±0.97	nd	nd	45.2±2.1c	51.1±3.9a	nd
	48h		nd	nd	274.9±19.8d	nd	nd	108.9±16.7f	61.6±4.7a	nd
	24h	SSF	4.56±0.65a	nd	730.4±14.9e	2.24±0.43a	32.1±1.9b	167.7±15.3g	487.4±14.9b	18.6±0.9c
	48h		6.12±0.99b	nd	814.8±16.8f	24.4±1.3b	137.9±5.2c	609.4±15.9h	458.6±15.1b	18.3±1.0c
***Levilactobacillus brevis* No. 173**	24h	SMF	nd	nd	92.3±7.1b	nd	nd	nd	53.9±4.3a	nd
	48h		nd	nd	95.7±4.2b	nd	1.32±0.41a	28.3±2.1b	54.4±4.0a	nd
	24h	SSF	5.84±0.86b	nd	833.4±17.0f	nd	nd	26.6±2.4b	516.2±15.7b	19.2±2.4c
	48h		5.55±0.79b	nd	854.7±16.2f	nd	nd	31.1±2.8b	518.7±15.6b	21.5±2.1c
***Leuconostoc mesenteroides* No. 225**	24h	SMF	nd	nd	96.1±4.7b	nd	nd	5.50±0.96a	49.7±3.5a	nd
	48h		nd	nd	99.9±4.9b	nd	nd	34.7±2.3b	52.4±4.6a	nd
	24h	SSF	5.06±0.88ba	nd	778.9±14.6f	1.93±0.97a	1.78±0.82a	85.4±3.83	460.2±15.3b	18.9±2.1c

	48h		5.32±0.86a,b	nd	784.5±18.1f	nd	nd	61.2±3.0d	468.9±13.9b	8.21±0.96b
***Liquorilactobacillus uvarum* No. 245**	24h	SMF	nd	nd	58.1±3.8a	nd	nd	0.99±0.09a	49.9±3.4a	nd
	48h		nd	nd	99.8±6.4b	nd	nd	17.7±1.4b	61.2±4.7a	1.52±0.08a
	24h	SSF	6.35±0.75b	nd	229.5±14.3c	nd	nd	22.0±1.2b	440.0±14.2b	17.2±1.8c
	48h		7.53±1.12c	nd	670.1±15.2e	nd	nd	25.2±2.1b	457.9±14.4b	18.1±1.7c

TRP – tryptamine; PHE - phenylethylamine; PUT - putrescine; CAD - cadaverine; HIS - histamine; TYR - tyramine; SPRMD - spermidine; SPRM – spermine; nd - not determined.

Data are represented as means (n = 6) ± standard error. [a-b] mean values denoted with different letters, indicates significantly different values between the lines ($p \leq 0.05$).
